# Surgical management of complex ileocolonic Crohn’s disease: a survey of IBD colorectal surgeons to assess variability in operative strategy

**DOI:** 10.1007/s00384-021-03892-z

**Published:** 2021-02-25

**Authors:** E Garofalo, F Selvaggi, A Spinelli, G Pellino, K Flashman, M Frasson, M Carvello, N de’Angelis, A Garcia-Granero, M Harper, J Warusavitarne, M Coleman, E Espin, V Celentano

**Affiliations:** 1 Department of General Surgery, Sant’Andrea Hospital, La Sapienza University, Rome, Italy; 2 Department of Advanced Medical and Surgical Sciences, Universitádella Campania “Luigi Vanvitelli”, Naples, Italy; 3 Department of Biomedical Sciences, Humanitas University, Via Rita Levi Montalcini 4, 20072 Pieve Emanuele, Milan, Italy; 4 IRCCS Humanitas Research Hospital -, via Manzoni 56, 20089 Rozzano, Milan, Italy; 5 Colon and Rectal Surgery Division, Humanitas University, Rozzano, Milan, Italy; 6 Colorectal Unit, Queen Alexandra Hospital – Portsmouth Hospitals NHS Trust, Portsmouth, UK; 7 Department of General Surgery, Colorectal Unit, La Fe University and Polytechnic Hospital, University of Valencia, Valencia, Spain; 8 Department of Digestive Surgery, Assistance Publique Hôpitaux de Paris, Henri Mondor Hospital, Université Paris-Est (UEP), Créteil, France; 9 Colorectal Surgery, Hospital Universitario Son Espases, Palma de Mallorca, Spain; 10 University of Portsmouth, Portsmouth, UK; 11 Department of Colorectal Surgery, St Mark’s Hospital, Harrow, Middlesex UK; 12 Department of Colorectal Surgery, University Hospitals Plymouth NHS Trust, Plymouth, UK; 13 Department of General Surgery, Hospital Valle de Hebron, Universitat Autonoma de Barcelona, Barcelona, Spain; 14 Department of Surgery and Cancer, Imperial College, London, UK

**Keywords:** Crohn’s disease, Laparoscopic surgery, Colorectal surgery, Ileocaecal resection, Inflammatory bowel disease

## Abstract

**Introduction:**

To explore the reported variability in the surgical management of ileocolonic Crohn’ s disease and identify areas of standard practice, we present this study which aims to assess how different colorectal surgeons with a subspecialty interest in inflammatory bowel disease (IBD) surgery may act in different clinical scenarios of ileocolonic Crohn’s disease.

**Methods:**

Anonymous videos demonstrating the small bowel walkthrough and anonymised patients’ clinical data, imaging and pathological findings were distributed to the surgeons using an electronic tool. Surgeons answered on operative strategy, bowel resections, management of small bowel mesentery, type of anastomosis and use of stomas.

**Results:**

Eight small bowel walkthrough videos were registered and 12 assessors completed the survey with a questionnaire completion rate of 87.5%. There was 87.7% agreement in the need to perform an ileocolonic resection. However, the agreement for the need to perform associated surgical procedures such as strictureplasties or further bowel resections was only 57.4%. When an anastomosis was fashioned, the side to side configuration was the most commonly used. The preferred management of the mesentery was dissection close to the bowel.

**Conclusions:**

The decision on the main procedure to be performed had a high agreement amongst the different assessors, but the treatment of multifocal disease was highly controversial, with low agreement on the need for associated procedures to treat internal fistulae and the use of strictureplasties. At the same time, there was significant heterogeneity in the decision on when to anastomose and when to fashion an ileostomy.

**Supplementary Information:**

The online version contains supplementary material available at 10.1007/s00384-021-03892-z.

## Introduction

Most patients with ileocolonic Crohn’s disease (CD) require surgical intervention during their lifetime, and in view of the relapsing course of the disease, a multidisciplinary team (MDT) approach [1] is mandatory. CD surgery is technically challenging, and variability may exist amongst colorectal surgeons in the adoption of different surgical procedures, such as extent of resection and use of bowel sparing techniques, need for diverting stomas or for management of internal fistulae and abscesses.

To explore this variability in the surgical management of ileocolonic CD and identify areas of standard practice, we present this study which aims to assess how different colorectal surgeons with a subspecialty interest in inflammatory bowel disease (IBD) surgery may act in different clinical scenarios of ileocolonic CD, with the primary objective to evaluate the inter-observer variability in the operative strategy in complex ileocolonic CD cases, with particular reference to surgical approach, extent and site of resections, use of bowel sparing techniques and diverting stomas.

## Methods

### Study setting

According to a previous study protocol [2], anonymous laparoscopic videos were recorded and edited at Queen Alexandra Hospital (Portsmouth, UK) to demonstrate the small bowel “walkthrough” in patients undergoing laparoscopic ileocolonic resection for primary and recurrent CD during the 3-month study period from March to May 2019. The small bowel walkthrough consisted of the entire exploration of the small bowel from the Treitz ligament to the ileocaecal valve or neo-terminal ileum, using a “hand over hand” technique with atraumatic forceps [2]. All procedures were performed by a single surgeon with expertise in IBD surgery in an attempt to mitigate the confounding factors of approach variation.

### Study design

Colorectal surgeons with expertise in minimally invasive surgery and IBD were selected as committee members to develop a survey on operative strategy for treatment of complex ileocolonic CD. Inclusion criteria for the committee members included evidence of previously published experience in CD surgery guideline development [3], distance learning in surgery [4], minimally invasive surgery training programme development and dissemination of online surgical videos [5]. Twelve experts made up this committee.

The committee identified items for inclusion in the survey by discussion through e-mails and face-to-face meetings. The final survey (Appendix 1) included items suggested by committee members as well as items adapted from the MREnterography or ulTRasound in Crohn’s disease (METRIC) study protocol [6] and from the classification of severity of mesenteric involvement described by Coffey et al. [7].

### Survey distribution

The anonymous videos demonstrating the small bowel walkthrough were distributed to the committee members together with the anonymous survey using an electronic tool (Enalyzer, Denmark, www.enalyzer.com). Anonymised patients’ clinical data, imaging and pathological findings were also shared with the committee members.

### Primary and secondary outcomes

The primary outcome was the rate of inter-observer variability in the operative strategy concerning type of surgical approach, extent and site of resections and strictureplasties and use of diverting stomas. The secondary outcomes were surgical preferences for management of the small bowel mesentery and anastomotic configuration.

### Statistical analysis

Categorical variables are presented as frequency or percentage and were compared with the use of the chi-squared test or Fisher’s exact test, as appropriate. Continuous variables are presented as mean (±standard deviation) or median (range) and were compared with the use of Student’s *t* test. The Mann-Whitney *U* test was used for continuous, not normally distributed outcomes. Inter-rater reliability was estimated by overall percent agreement and by Fleiss’ kappa (*ĸ*) [8] along with its 95% confidence interval. *ĸ* values from 0.21 to 0.4 were considered as indicating fair agreement, while values from 0.41 to 0.6 and >0.61 were considered as indicating moderate and substantial agreement, respectively.

## Results

Eight small bowel walkthrough videos (Appendix 2) were distributed and 12 assessors completed the survey. Out of the 672 expected answers, 588 were returned, with a questionnaire completion rate of 87.5%. The quality of the small bowel walkthrough was widely considered acceptable, with assessors being unable to comment on the case only in 18 out of 588 questions (3%).

### Surgical resection and associated surgical procedures

There was an agreement of 87.7% amongst the assessors in the need to perform an ileocolic resection (*k*= 0.82; 95% CI 0.64, 0.99). However, the agreement for the need to perform associated surgical procedures such as strictureplasties, further bowel resections or repair of internal fistulae was quite low, with an agreement of 57.4% (*k*=0.36; 95% CI 0.13, 0.59). Amongst all the assessors, only 1 suggested conversion to open surgery in one of the procedures (estimated conversion rate of 1.04%). The full results of the survey are shown in Table 1.

**Table 1:** Assessors’ agreement for the need to perform any surgical procedure

CASE	MAIN PROCEDURE		UNABLE TO COMMENT		OTHER ASSOCIATED PROCEDURES				
	Ileocaecal resection (%)	No resection (%)	Conversion to open (%)		Stricturoplasty (%)	Other bowel resection (%)	Bladder resection (%)	Primary repair of colon (%)	Abscess Debridement (%)
1	90.9	0.	0.	9.1	27.3	0.	0.	0.	0.
2	88.9	0.	0.	11.1	0.	22.2	0.	22.2	0.
3	100	0.	0.	0.	0.	0.	0.	0.	0.
4	100	0.	0.	0.	0.	66.7	0.	22.2	11.1
5	91.7	0.	8.3	8.3	0.	0.	0.	0.	0.
6	100	0.	0.	0.	0.	0.	9.1	0.	27.3
7	72.7	27.3	0.	0.	72.7	0.	0.	0.	0.
8	100	0.	0.	0.	45.4	0.	0.	0.	0.

### Differences in anastomosis formation and defunctioning stoma

There was significant heterogeneity in the decision to fashion or not an anastomosis, with an agreement of 70.4% (*k* = 0.32; 95% CI 0.08, 0.56). When the anastomosis was not fashioned, there were reported differences in the management of the distal colonic end; however, a double-barrelled stoma (76.4%) was the preferred option over a closed intra-abdominal stump (11.8%) or an ileocolic anastomosis protected by a loop ileostomy (11.8%). When an anastomosis was fashioned, the side to side anastomosis was the most commonly used as detailed in Appendix 3.

### Surgical management of the mesentery

There was significant heterogeneity in the management of the mesentery, with an overall agreement of 45.6% (*k*= 0.28; 95% C.I. 0.15–0.40) with a slight preference for resection of the mesentery close to the bowel (33.3 to 77.8% of the cases) rather than radical cancer-like resection (0 to 41.7%) or resection of macroscopically affected mesentery only ( 0 to 25%).

## Discussion

Our study explored the variability in the intraoperative strategy in patients undergoing CD surgery, by evaluating via an anonymous survey the differences in the suggested surgical approach amongst a group of colorectal surgeons with subspecialty interest in IBD and expertise in minimally invasive surgery. The assessors were fully informed of the preoperative data of the patients, including imaging results and were acknowledged of the intraoperative findings by edited videos demonstrating the laparoscopic small bowel walkthrough. The decision on the main procedure to be performed, ileocolonic resection, had a high agreement amongst the different assessors; however, how to treat associated disease was highly controversial, with very low agreement on the need for associated procedures to treat internal fistulae or use of strictureplasties. At the same time, the decision on when to anastomose and when to fashion an ileostomy was quite subjective, with different techniques suggested for the management of the colonic stump, which was left intra-abdominally as a closed end by some surgeons or brought out as a mucus fistula or double-barrelled stoma by the majority. Concerning the extent of mesenteric resection, a non-radical resection seemed the most common approach in our cohort, probably because of concerns regarding haemorrhagic dangers associated with division of the mesentery in patients with CD and potential need for increased length of resected bowel if larger mesenteric segments are removed [9].

Our study introduced for the surgeons-assessors the virtual scenario of deciding on the surgical strategy based on the preoperative information and a limited view of the macroscopic bowel assessment obtained at laparoscopy. The lack of direct involvement in the preoperative discussion with patient and his/her family members creates an artificial scenario where decisions may not be tailored to the patients’ preferences. Nevertheless, we gave the exact same information to all the blind assessors, who advised on the CD management on the basis of their knowledge and experience for the patient’s best outcome. We must acknowledge this reported variation in preferred surgical approaches and outcomes [10] which could reflect lack of standardisation. The results of our study strengthen the role of video-based education and tele-mentoring to overcome the reported limited surgical trainees’ exposure to these complex procedures [11]. Surgeons could share video recordings of intraoperative findings in dedicated teaching sessions of multi-institutional meetings, with the aim to enhance surgical training in IBD and facilitate standardisation of surgical techniques.

Unfortunately, we did not include the Kono-S anastomotic technique amongst the options for anastomosis configuration, and therefore the use of the Kono-S anastomosis by the assessors has not been evaluated, despite the survey allowing free text insertion for additional comments from the assessors. Nevertheless, it should be noted that the Kono-S anastomosis is still performed in a small proportion of patients, which was 2.3% according to a recent study reporting on 427 patients undergoing ileocolonic resection for CD [12]. Another obvious limitation of the study is the lack of tactile feedback, which significantly limited the opportunity for the assessors to identify bowel segments requiring additional interventions, explaining why the use of bowel sparing techniques such as strictureplasties was minimal, despite the assessors being fully aware of the preoperative MRI imaging. This lack of bowel sparing techniques reflects not only a selection bias in the videos edited for presentation to the assessors but also the difficulties in making a thorough assessment of the small bowel involvement at laparoscopy, where a trusty agreement may exists amongst surgeons on the presence of bowel thickening and mesenteric fat wrapping, but not on the evaluation of proximal lesions and severity of mesenteric disease. [2]

## Conclusions

Our study reported high agreement amongst IBD surgeons on the main procedure to be performed in selected cases of ileocolonic CD, but the treatment of multifocal disease was highly controversial, with very low agreement on the need for associated procedures to treat internal fistulae and the use of strictureplasties. At the same time, there was significant heterogeneity in the decision on when to anastomose and when to fashion an ileostomy.

## Supplementary Information

ESM 1 Imaging and surgical findings of the 8 video-recorded cases (DOCX 1120 kb)

ESM 2 Assessors’ agreement for the need to perform anastomosis and/or defunctioning (DOCX 15 kb)

ESM 3 Assessors’ agreement on the anastomosis configuration (DOCX 16 kb)
