# Correction to: Surgical management of complex ileocolonic Crohn’s disease: a survey of IBD colorectal surgeons to assess variability in operative strategy

**DOI:** 10.1007/s00384-021-03942-6

**Published:** 2021-05-07

**Authors:** E Garofalo, F Selvaggi, A Spinelli, G Pellino, K Flashman, M Frasson, M Carvello, N de’Angelis, A Garcia-Granero, M Harper, J Warusavitarne, M Coleman, E Espin, V Celentano

**Affiliations:** 1 Department of General Surgery, Sant’Andrea Hospital, La Sapienza University, Rome, Italy; 2 Department of Advanced Medical and Surgical Sciences, Universitádella Campania “Luigi Vanvitelli”, Naples, Italy; 3 Department of Biomedical Sciences, Humanitas University, Via Rita Levi Montalcini 4, 20072 Pieve Emanuele, Milan, Italy; 4 IRCCS Humanitas Research Hospital, via Manzoni 56, 20089 Rozzano, Milan, Italy; 5 Colon and Rectal Surgery Division, Humanitas University, Rozzano, Milan, Italy; 6 Colorectal Unit, Queen Alexandra Hospital – Portsmouth Hospitals NHS Trust, Portsmouth, UK; 7 Department of General Surgery, Colorectal Unit, La Fe University and Polytechnic Hospital, University of Valencia, Valencia, Spain; 8 Department of Digestive Surgery, Assistance Publique Hôpitaux de Paris, Henri Mondor Hospital, Université Paris-Est (UEP), Créteil, France; 9 Colorectal Surgery, Hospital Universitario Son Espases, Palma de Mallorca, Spain; 10 University of Portsmouth, Portsmouth, UK; 11 Department of Colorectal Surgery, St Mark’s Hospital, Harrow, Middlesex, UK; 12 Department of Colorectal Surgery, University Hospitals Plymouth NHS Trust, Plymouth, UK; 13 Department of General Surgery, Hospital Valle de Hebron, Universitat Autonoma de Barcelona, Barcelona, Spain; 14 Department of Surgery and Cancer, Imperial College, London, UK

**Correction to: International Journal of Colorectal Disease**

10.1007/s00384-021-03892-z

In the original published version of this article, the co-author correct **A Spinelli’s** affiliation has been added in the list of affiliations.

The original article has been corrected.

