# Supplementary material for: Surgical management of complex ileocolonic Crohn’s disease: a survey of IBD colorectal surgeons to assess variability in operative strategy
Source: Int J Colorectal Dis. 2021 Feb 25;36(8):1811–5. doi: 10.1007/s00384-021-03892-z (PMC8279976; doi:10.1007/s00384-021-03892-z)

**VIDEO-LAPAROSCOPIC ASSESSMENT OF THE SMALL BOWEL IN CROHN’S DISEASE :** the distribution of anonymous videos demonstrating the small bowel walkthrough together with the anonymous survey using www.enalyzer.com.


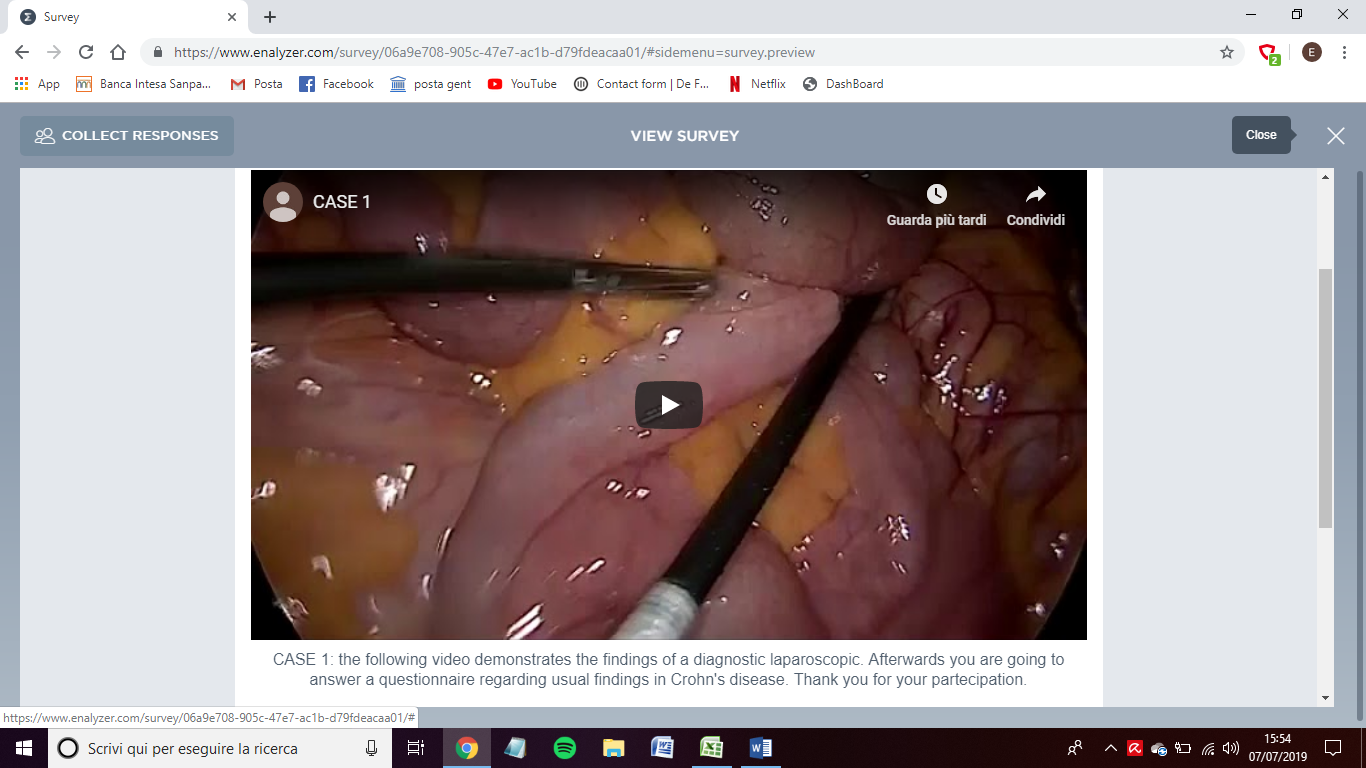


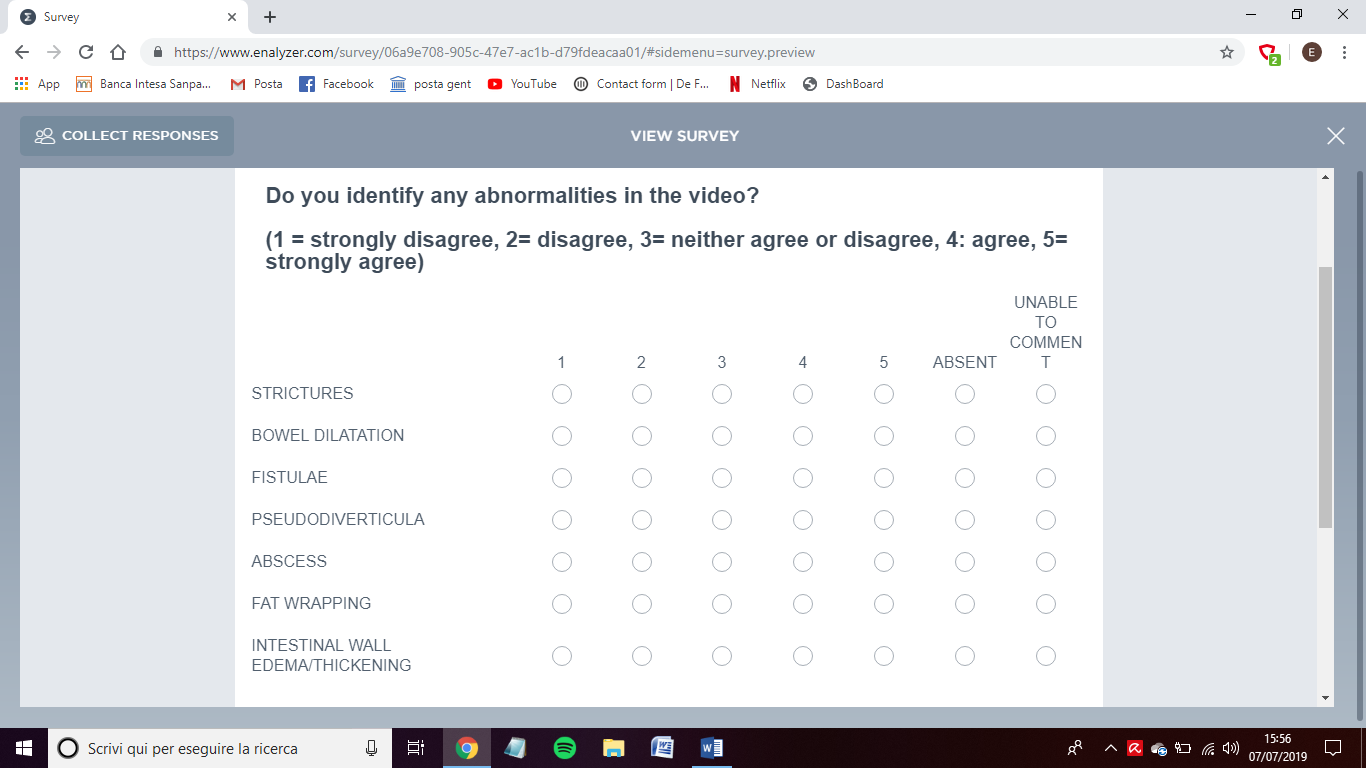


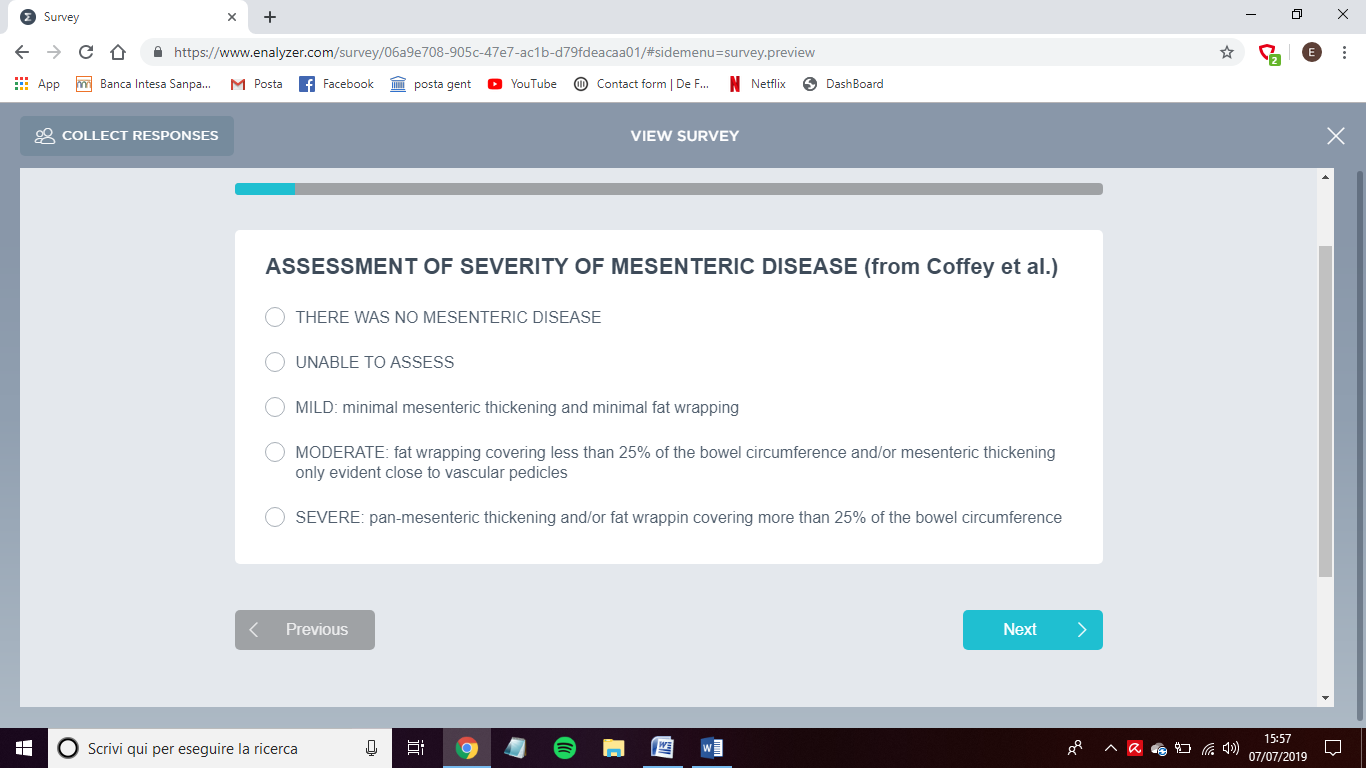


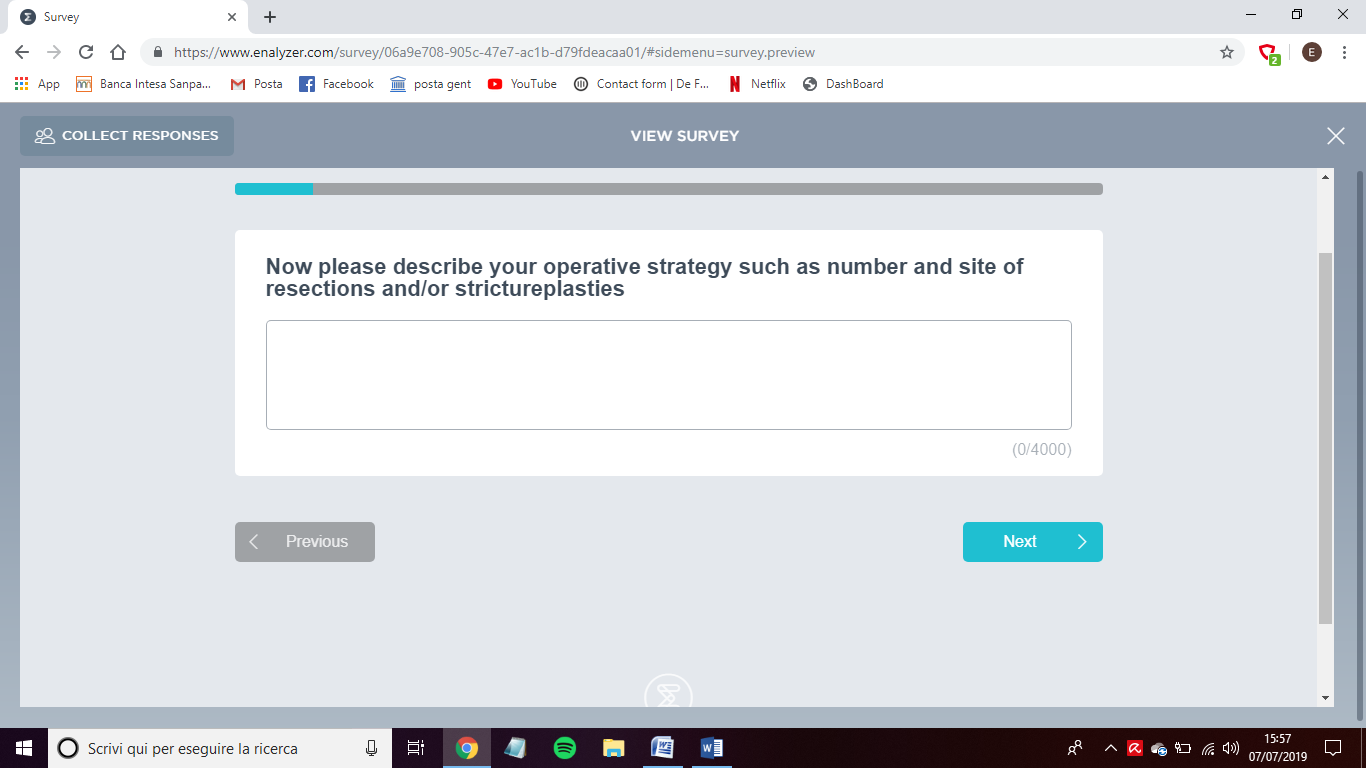


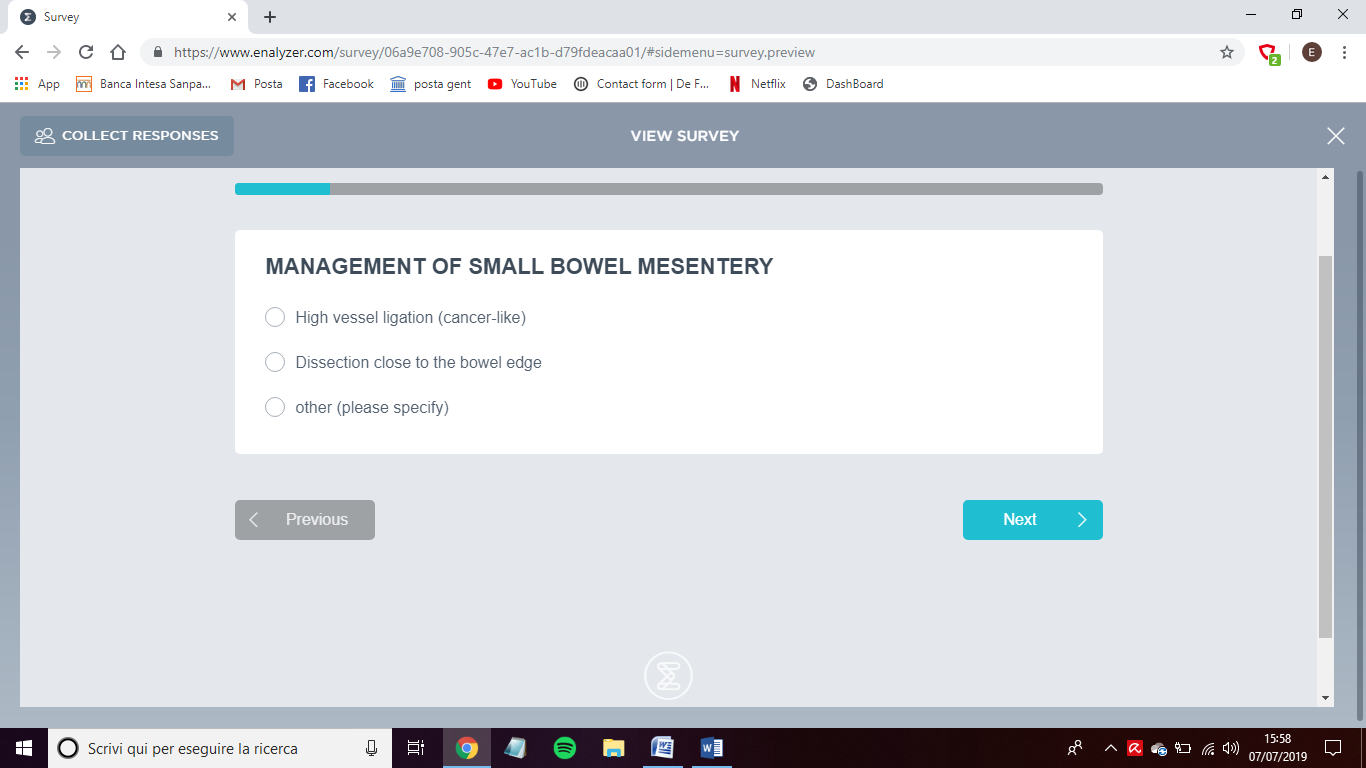


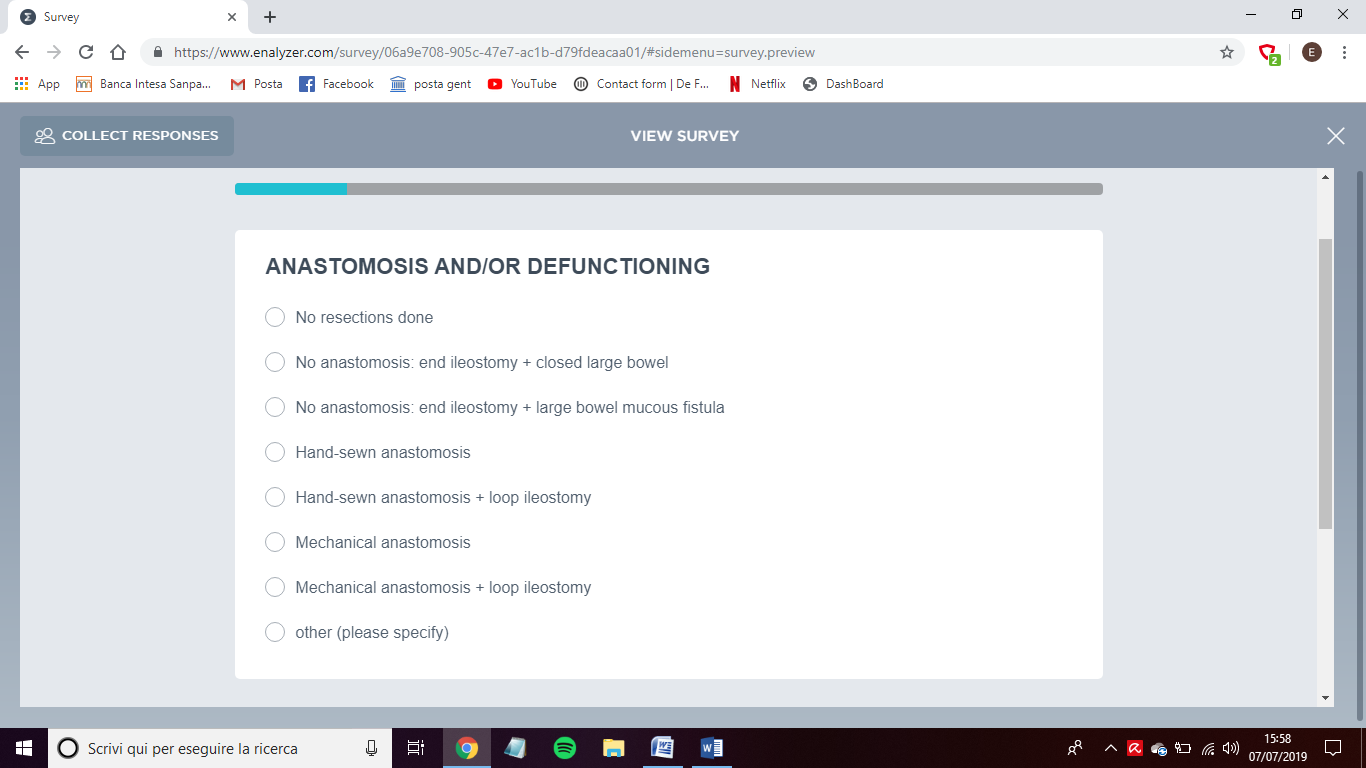


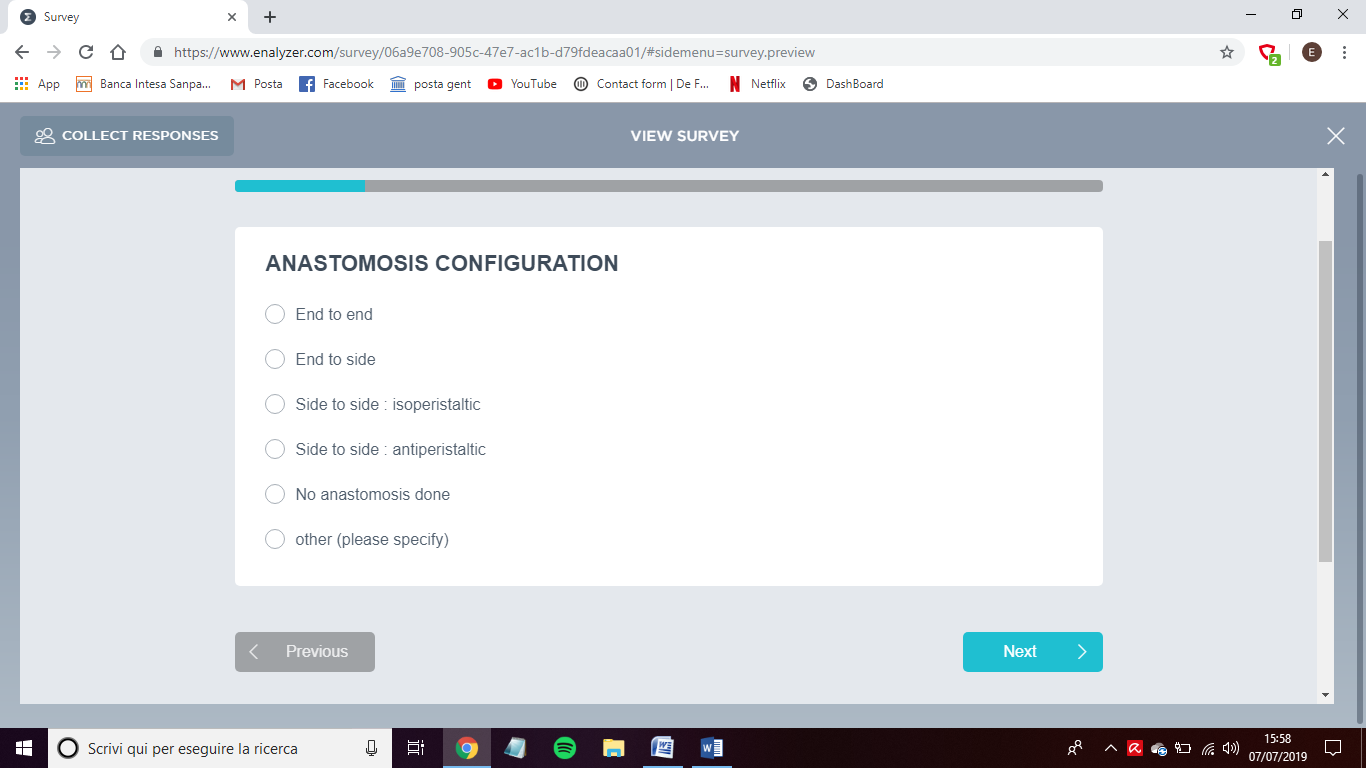

Supplement: Supplementary file 1 — Imaging and surgical findings of the 8 video-recorded cases (DOCX 1120 kb) [file 384_2021_3892_MOESM1_ESM.docx]
