# Supplementary material for: Surgical management of complex ileocolonic Crohn’s disease: a survey of IBD colorectal surgeons to assess variability in operative strategy
Source: Int J Colorectal Dis. 2021 Feb 25;36(8):1811–5. doi: 10.1007/s00384-021-03892-z (PMC8279976; doi:10.1007/s00384-021-03892-z)
Supplement: Supplementary file 2 — Assessors’ agreement for the need to perform anastomosis and/or defunctioning (DOCX 15 kb) [file 384_2021_3892_MOESM2_ESM.docx]

| **CASE (N)** | **PREOPERATIVE IMAGING** | **SURGICAL FINDINGS** |
| --- | --- | --- |
| 1 | Short stricture of the terminal ileum over 5 cm with mild upstream small bowel dilatation. | 5 cm stricture at terminal ileum with mild pre-stenotic dilatation. No fat wrapping. |
| 2 | 20 cm of active inflammation at the terminal ileum which is in close proximity with the sigmoid colon. | 15cm of terminal ileitis with ileo-sigmoid fistula and psoas abscess. |
| 3 | 10 cm stricture at the neo-terminal ileum. Fat hypertrophy with upstream bowel dilatation | Recurrent crohn's disease with stricture at the anastomosis (10 cm of neo-terminal ileum) and upstream small bowel dilatation. |
| 4 | 20 cm of distal ileum thickening with evidence of stricturing. Pre-stenotic dilatation of the ileum. Fistula to the sigmoid colon. | Penetrating crohn's disease of the terminal ileum (20 cm) with ileo-sigmoid fistula. |
| 5 | Active Crohn’s disease involving the distal 30 cm of terminal ileum with a concomitant para-caecal abscess of 5 cm. | Perforated terminal ileum (35 cm) with abscess in the terminal ileum mesentery. |
| 6 | Transmural inflammation of the terminal ileum. Abdominal wall collection measuring 4.6 cm abutting the inflamed ileum and containing a gas bleb suggesting fistulation to the bowel. | Penetrating crohn's disease of the terminal ileum (with severe fat wrapping) with fistula to the abdominal wall and abdominal wall abscess. |
| 7 | Two short strictures in the distal ileum (35cm from the ileo-caecal junction). Also another mid ileum stricture of 3 cm. Moderate pre-stenotic dilatation of the small bowel. | Several strictures in the proximal ileum over 30 cm with moderate fat wrapping.  Another 15 cm stricture in the distal ileum with mild fat wrapping. |
| 8 | 5 cm segment of active inflammatory disease involving the ileo-caecal junction. Separate 20 cm segment of active disease within the distal jejunal/proximal ileum. Mild prestenotic dilatation. | 40 cm of inflamed distal jejunum/proximal ileum. No distal ileum disease |

Appendix 2.. Imaging and surgical findings of the 8 video-recorded cases.
