# Supplementary material for: Surgical management of complex ileocolonic Crohn’s disease: a survey of IBD colorectal surgeons to assess variability in operative strategy
Source: Int J Colorectal Dis. 2021 Feb 25;36(8):1811–5. doi: 10.1007/s00384-021-03892-z (PMC8279976; doi:10.1007/s00384-021-03892-z)
Supplement: Supplementary file 3 — Assessors’ agreement on the anastomosis configuration (DOCX 16 kb) [file 384_2021_3892_MOESM3_ESM.docx]

|  | **CASE 1** | **CASE 2** | **CASE 3** | **CASE 4** | **CASE 5** | **CASE 6** | **CASE 7** | **CASE 8** |
| --- | --- | --- | --- | --- | --- | --- | --- | --- |
| **NO ANASTOMOSIS DONE (%)** | 9.1 | 11.1 | 0 | 22.2 | 83.3 | 45.5 | 36.4 | 0 |
| **END TO SIDE** | 18.2 | 11.1 | 11.1 | 11.1 | 8.3 | 9.1 | 9.1 | 9.1 |
| **SIDE TO SIDE**  Isoperistaltic (%):  Antiperistaltic (%): | 36.4  36.4 | 33.3  33.3 | 55.6  33.3 | 33.3  33.3 | 8.3  0 | 36.4  9.1 | 27.3  27.3 | 45.4  45.5 |
| **END TO END (%)** | 0 | 11.1 | 0 | 0 | 0 | 0 | 0 | 0 |

Appendix 3. Assessors’ agreement on the anastomosis configuration
